# Grain yield trade-offs in spike-branching wheat can be mitigated by elite alleles affecting sink capacity and post-anthesis source activity

**DOI:** 10.1093/jxb/erad373

**Published:** 2023-09-22

**Authors:** Ragavendran Abbai, Guy Golan, C Friedrich H Longin, Thorsten Schnurbusch

**Affiliations:** Research Group Plant Architecture, Leibniz Institute of Plant Genetics and Crop Plant Research (IPK), OT Gatersleben, 06466 Seeland, Germany; Research Group Plant Architecture, Leibniz Institute of Plant Genetics and Crop Plant Research (IPK), OT Gatersleben, 06466 Seeland, Germany; State Plant Breeding Institute, University of Hohenheim, Fruwirthstr. 21, 70599 Stuttgart, Germany; Research Group Plant Architecture, Leibniz Institute of Plant Genetics and Crop Plant Research (IPK), OT Gatersleben, 06466 Seeland, Germany; Martin Luther University Halle-Wittenberg, Faculty of Natural Sciences III, Institute of Agricultural and Nutritional Sciences, 06120 Halle, Germany; John Innes Centre, UK

**Keywords:** Grain number, grain weight, grain yield, inflorescence branching, QTLs, senescence rate, source–sink strength, trade-offs

## Abstract

Introducing variations in inflorescence architecture, such as the ‘Miracle-Wheat’ (*Triticum turgidum* convar. *compositum* (L.f.) Filat.) with a branching spike, has relevance for enhancing wheat grain yield. However, in the spike-branching genotypes, the increase in spikelet number is generally not translated into grain yield advantage because of reduced grains per spikelet and grain weight. Here, we investigated if such trade-offs might be a function of source–sink strength by using 385 recombinant inbred lines developed by intercrossing the spike-branching landrace TRI 984 and CIRNO C2008, an elite durum (*T. durum* L.) cultivar; they were genotyped using the 25K array. Various plant and spike architectural traits, including flag leaf, peduncle, and spike senescence rate, were phenotyped under field conditions for 2 consecutive years. On chromosome 5AL, we found a new modifier QTL for spike branching, *branched head*^*t*^*3* (*bh*^*t*^*-A3*), which was epistatic to the previously known *bh*^*t*^*-A1* locus. Besides, *bh*^*t*^*-A3* was associated with more grains per spikelet and a delay in flag leaf senescence rate. Importantly, favourable alleles, viz. *bh*^*t*^*-A3* and *grain protein content* (*gpc-B1*) that delayed senescence, are required to improve grain number and grain weight in the spike-branching genotypes. In summary, achieving a balanced source–sink relationship might minimize grain yield trade-offs in Miracle-Wheat.

## Teaser

Elite *branched head*^*t*^*3* (*bh*^*t*^*-A3* ) and *grain protein content* (*gpc-B1* ) alleles mitigate grain yield trade-offs in spike branching wheat by prolonging post-anthesis source activity.

## Introduction

The wheat (*Triticum* spp.) inflorescence or ‘spike’ is a determinate structure harbouring the grain-bearing spikelets on its rachis in a distichous pattern. During immature spike development, the inflorescence meristem gives rise to multiple spikelet meristems in an acropetal manner. In turn, each spikelet meristem (indeterminate) produces florets, which potentially form grains (Kirby and Appleyard, 1984; Koppolu and Schnurbusch, 2019). However, some exceptions deviate from this standard developmental programme, such as the ‘Miracle-Wheat’ that produces a non-canonical spike with lateral branches instead of spikelets. In the case of *Triticum turgidum* convar. *compositum* (L.f.) Filat. accessions, the spikelet meristems lose their identity and determinacy while partially behaving as inflorescence meristems, producing lateral branches or multiple spikelets per rachis node, due to mutation in the DNA-binding domain of the *branched head*^*t*^ (*bh*^*t*^) allele, encoding an APETALA2/ETHYLENE RESPONSIVE FACTOR (AP2/ERF) transcription factor (Poursarebani *et al.*, 2015). Similarly, in hexaploid wheat, variations for supernumerary spikelet formation were also found for the wheat genes *FRIZZY PANICLE* (*WFZP*) (Dobrovolskaya *et al.*, 2015), *Photoperiod-1* (*Ppd-1*) (Boden *et al.*, 2015), *TEOSINTE BRANCHED1* (*TB1*) (Dixon *et al.*, 2018), and *HOMEOBOX DOMAIN-2* (*HB-2*) (Dixon *et al.*, 2022). While branching spikes have considerably higher yield potential, i.e. higher spikelet number, they often suffer from grain weight trade-offs, as observed in the tetraploid Miracle-Wheat (Poursarebani *et al.*, 2015). Moreover, despite the increase in overall grain number per spike, spikelet fertility (grains per spikelet) decreased in response to spike branching (Wolde *et al.*, 2021).

A large body of evidence suggests that wheat grain yield is an outcome of multiple trait–trait interactions mediated by developmental, physiological, and environmental factors across the entire lifespan, although some stages are more critical than others (Guo *et al.*, 2016, 2017, 2018a, b; Brinton and Uauy, 2019; Reynolds *et al.*, 2022; Murchie *et al.*, 2023; Slafer *et al.*, 2023). They can broadly be classified as source and sink strength related, which jointly determine a particular genotype’s assimilate production and reallocation potential. Typically, green tissues of the plant—both foliar (leaves) and non-foliar (peduncle, spikes)—are the photosynthesizing organs that act as ‘source’ for resource generation (Molero and Reynolds, 2020; Chang *et al.*, 2022). In the pre-anthesis phase, assimilates are partitioned to both vegetative biomass establishment and developing spikes—which determine the overall yield potential (Slafer, 2003; Fischer, 2011). The inflorescence architecture, viz. spikelet number per spike, floret number per spikelet, carpel size, rachis length, etc., are determined before anthesis (Kirby and Appleyard, 1984; Brinton and Uauy, 2019). For instance, the ovary size during flowering regulated floret and grain survival in a panel of 30 wheat genotypes (Guo *et al.*, 2016). Likewise, the duration of leaf initiation, spikelet initiation, and stem elongation period influenced spike fertility in bread wheat (Roychowdhury *et al.*, 2023). The source strength is often characterized by radiation use efficiency, i.e. the ability to intercept light and biomass production (Acreche and Slafer, 2009; Molero *et al.*, 2019). However, the balance between the resources allocated to the ‘vegetative’ versus ‘reproductive’ tissues largely dictates the yield potential (Ferrante *et al.*, 2013; Dreccer *et al.*, 2014), a trait that has been under selection throughout the history of wheat breeding. The deployment of semi-dwarf *Rht-1* alleles (‘green revolution’ gene) significantly increased the harvest index and the grain number per unit area, possibly by enhancing the flow of assimilates (as the stem length is considerably reduced) to the juvenile spikes (Fischer and Stockman, 1986; Slafer *et al.*, 2023). However, other strategies might currently be required to further the resource allocation to early spike development as the semi-dwarf *Rht-1* allele is already a selection target (Peng *et al.*, 1999). Increasing the harvest index in the genotypes with high biomass (more robust source) might enhance grain yield (Sierra-Gonzalez *et al.*, 2021). Overall, the source strength from the terminal spikelet stage to anthesis majorly determines grain number and size in wheat.

After anthesis, the initiation of senescence in the foliar, but also non-foliar tissues drives extensive remobilization of resources into the developing grains. Previous studies have indicated that flag leaf and spike photosynthesis contribute to most of the assimilates during the grain filling phase (Distelfeld *et al.*, 2014; Molero and Reynolds, 2020). Hence, a delayed senescence resulted in extended photosynthesis (functional stay-green), leading to higher grain yield (Kichey *et al.*, 2007; Christopher *et al.*, 2016; Chapman *et al.*, 2021b; Hassan *et al.*, 2021; Li *et al.*, 2022; Niu *et al*., 2023; Yan *et al*., 2023). However, the effect of delayed senescence was not consistent; for instance, prolonged photosynthesis influenced grain yield attributes only under low nitrogen conditions (Gaju *et al.*, 2011; Derkx *et al.*, 2012). The *GPC-B1* locus encoding NO APICAL MERISTEM (NAM), a NAC transcription factor, is the major regulator of senescence rate in wheat (Uauy *et al.*, 2006), but despite a 40% increase in flag leaf photosynthesis, the *NAM* RNAi wheat lines had no advantage in grain weight compared with the control plants (Borrill *et al.*, 2015). In addition, the stay-green phenotype of *gpc-A1* and *gpc-D1* mutants did not influence grain yield determinants (Avni *et al.*, 2014). However, Chapman *et al.* (2021b) reported that a novel *NAM-1* allele that delayed senescence was associated with a 14% increase in the final grain weight, possibly by enhancing resource remobilization. A plausible explanation for such discrepancies might be that grain yield in wheat is largely sink-limited (Reynolds *et al.*, 2005; Lichthardt *et al.*, 2020). The surplus water-soluble carbons that remain in the stem at physiological maturity supports this hypothesis (Serrago *et al.*, 2013). Thus, a higher sink capacity might be essential to capitalize on the extended photosynthetic period during the grain filling phase (Lichthardt *et al.*, 2020). In this context, a reductionist approach that focuses on characterizing individual component traits might assist in the deeper understanding of source–sink dynamics but also be integrated to pinpoint favourable combinations of alleles/haplotypes for improving wheat grain yield (Brinton and Uauy, 2019; Reynolds *et al.*, 2022).

As Miracle-Wheat has a stronger sink (higher spikelet number), we hypothesized that delimited post-anthesis source strength might explain the spike-branching-induced grain yield trade-offs. To examine this, we developed a biparental wheat population comprising about 385 recombinant inbred lines (RILs) by crossing the spike branching TRI 984 with an elite durum CIRNO C2008. The idea was to evaluate this population under field conditions for various architectural traits, as well as the senescence rate of the flag leaf, the peduncles, and the spike (details are in ‘Materials and methods’). In summary, our current study aims to explain (i) the relationship between senescence rate and trade-offs regulating grain yield (spike branching, grain number, grain weight); (ii) the underlying genetics of such trade-offs; (iii) favourable trait and allele combinations of relevant QTLs to balance grain yield trade-offs; and finally (iv) verify if spike branching might be a potential selection target to enhance grain yield in wheat.

## Materials and methods

### Population development

A biparental population comprising 385 RILs was developed by crossing the spike-branching Miracle-Wheat accession ‘TRI 984’ and elite durum from CIMMYT, ‘CIRNO C2008’ (hereafter referred to as ‘CIRNO’). A modified speed breeding method (Ghosh *et al.*, 2018; Watson *et al.*, 2018) was used for rapid generation advancement from F_3_ to F_5_. Initially, the grains were sown in the 96-well trays and grown in standard long day conditions, viz. 16 h light (19 °C) and 8 h dark (16 °C) for about 2 weeks. Later, the trays were transferred to speed breeding conditions viz. 22 h light (22 °C) and 2 h dark (17 °C) to accelerate growth. The spikes were harvested at maturity, and a similar method was used for the next cycle. Finally, the obtained F_5_ plants were multiplied under field conditions during the spring of 2020, and the resulting F_6_ grains (RILs) were genotyped and phenotyped.

### Genotyping and linkage map construction

The parental lines and three F_6_ grains per RIL were sown in 96-well trays and were grown in standard greenhouse conditions (16 h light (19 °C) and 8 h dark (16 °C)) for about 2 weeks. Leaves were sampled at the two-leaf stage from all the seedlings and stored at −80 °C until further use. During the sampling, the leaves from the three replications of a particular RIL were pooled, and genomic DNA was extracted. The DNA integrity was evaluated on an agarose gel, after which about 50 ng μl^−1^ aliquots were prepared for the genotyping. Eventually, the 25K wheat SNP array from SGS-TraitGenetics GmbH was used for genotyping the 385 RILs along with the parental lines. However, only the 21K markers scored to the A and B sub-genome were considered for further analysis; we found that 5089 makers were polymorphic (Supplementary Fig. S1A). A linkage map was developed using the regression and maximum likelihood methods in JoinMap v4.1 (Stam, 1993). A subset of 2128 markers was prepared after filtering, viz. without segregation distortion (determined based on a chi-square test), <10% heterozygosity, and <10% missing (Supplementary Fig. S1B, C). Haldane’s mapping function was used in the regression method, while the maximum likelihood method involved the spatial sampling thresholds of 0.1, 0.05, 0.03, 0.02, and 0.01 with three optimization rounds per sample. Outcomes from both these methods were used to determine the 14 linkage groups and final map order.

### Examining plant and spike architectural traits

#### Greenhouse conditions

After collecting the leaves for genotyping (as described in the previous section), the 2-week-old seedlings were vernalized at 4 °C for 1 month. Then, the seedlings were transferred to 9 cm square pots, grown in long day conditions (16 h light (19 °C) and 8 h dark (16 °C)). In both the parents—TRI 984 and CIRNO—tillers per plant (at booting) and spike number per plant (at maturity) were phenotyped. Besides, flag leaf verdancy was measured at eight different locations along the leaf blade (Borrill *et al.*, 2019) at heading and also 30 d after heading using the SPAD-502 chlorophyll meter (Konica Minolta). Standard fertilization was performed, and plants were treated with pesticides based on requirement.

#### Field conditions

The genotypes were screened at IPK-Gatersleben (51°49ʹ23″N, 11°17ʹ13″E, 112 m altitude) under field conditions for two growing seasons, viz. the F_6_-derived RILs in the spring of 2021 and the F_7_-derived RILs in the spring of 2022. They were grown in an α-lattice design with three replications, while each 1.5 m^2^ plot had six 20 cm-spaced rows comprising two genotypes (three rows each). Standard agronomic and management practices were in place throughout the growth cycle; however, the experimental trial was completely rainfed. Besides, a subset of genotypes (F_6_-derived RILs) in one replication was evaluated at the University of Hohenheim (48°42ʹ50″N, 9°12ʹ58″E, 400 m altitude) in 2022.

Plants from the inner rows (at least five measurements per plot) were considered for all the phenotyping except for grain yield per metre row, where the mean of all three rows of a particular genotype was measured. Days to heading (DTH) was determined at ‘Zadoks 55’, i.e. when half of the spike has emerged (Zadoks *et al.*, 1974) in about 50% of the plants in a particular plot. Later, this was converted into growing degree days (GDD) to account for temperature gradients (Miller *et al.*, 2001). The distance from the tip of the flag leaf to its base was considered as the flag leaf length, while the flag leaf width was the end-to-end horizontal distance at the middle of the leaf. At heading, flag leaf verdancy was measured using the SPAD-502 chlorophyll meter (Konica Minolta) at eight different locations along the leaf blade (Borrill *et al.*, 2019). Flag leaf senescence was screened 30 d after heading using a four-point severity scale from ‘1’ indicating the least senescence to ‘4’ for the highest senescence (Supplementary Fig. S2A). The number of senesced peduncles per 10 peduncles was counted from the inner rows to determine peduncle senescence (%) (Chapman *et al.*, 2021a). In this context, we found a gradient of yellowness in the peduncle across the RILs; however, in the current study, this was not differentiated, i.e. we had only two classes—green and yellow (Supplementary Fig. S2B). Days to maturity (DTM) was determined when most spikes turned yellow in a particular plot; later, this was converted to growing degree days similar to days to heading.

Spike weight, spike length (without awns), and straw biomass (dry weight of culm along with leaves) were measured after harvest. In addition, a scoring method was developed for estimating supernumerary spikelets (two spikelets per rachis node) and spike branching (true branching with mini-spikes from the rachis nodes) (Supplementary Fig. S3): ‘0’ (standard spike), ‘1’ (supernumerary spikelets only at the basal part of the spike), ‘2’ (supernumerary spikelets until half of the spike), ‘3’ (supernumerary spikelets throughout the spike), and ‘4’ (proper branching). Floret number was measured from the non-branching genotypes from two spikelets at the centre of the spike at harvest. Besides, derived traits such as grains per spikelet, grain filling duration (Chapman *et al.*, 2021a), and harvest index were calculated as follows:


$$\begin{array}{l} Grains\;per\;spikelet=\frac{Grains\;per\;spike}{Spikelet\;number\;per\;spike} \end{array}$$

$$\begin{array}{l} Grain\;f\;illing\;duration\;=Days\;to\;maturity-Days\;to\;heading \end{array}$$

$$\begin{array}{l} Harvest\;index=\frac{Grain\;weight\;per\;spike}{Straw\;biomass+Spike\;weight} \end{array}$$

The ‘Marvin’ digital grain analyser (GTA Sensorik GmBH, Neubrandenburg, Germany) was used to determine grains per spike, thousand-grain weight, grain length, and grain width. We also recorded the grain width and length of the parental lines manually using a Vernier calliper to reconfirm the observed trend from the ‘Marvin’ digital seed analyser (Supplementary Fig. S4). All the above-mentioned traits were recorded at IPK-Gatersleben, while only the spike architectural traits were phenotyped from the experiment conducted at the University of Hohenheim.

### Phenotypic and genetic analyses

Genstat 19 (VSN International, Hemel Hempstead, UK) and GraphPad Prism 9.3.1 (GraphPad Software, Boston, MA, USA) were used for all the statistical analyses. Ordinary one-way ANOVA followed by Dunnett’s multiple comparisons test was employed for multiple-range comparisons, and Student’s unpaired *t*-test was used to compare two groups. Pearson’s correlation was used to study the relationship among the traits of interest; besides, simple linear regression assisted in understanding the effect of a particular trait (explanatory variable) on another (response variable). The corresponding figures contain all the relevant details, such as *P*-value, *R*^2^, and the number of samples compared; Supplementary Datasets S1 and S2 contain the source data used for statistical analysis.

QTL mapping was performed in Genstat 19 using the following criteria: (i) step size of 10 cM, (ii) minimum cofactor proximity of 50 cM, (iii) minimum QTL separation distance of 30 cM, and (iv) genome wide significance, α=0.05. Simple interval mapping was performed as an initial scan to determine the positions of potential candidate QTL(s). These positions were used as cofactors for multiple rounds of composite interval mapping (CIM); CIM was repeated until similar results were obtained at least three consecutive times. Finally, QTL backward selection was carried out after CIM to estimate various QTL effects, including the determination of QTL interval, high-value allele, additive effects, and phenotypic variance explained. The QTLs were then visualized using MapChart 2.32 (Voorrips, 2002).

## Results

### Spike branching affects grains per spikelet and thousand-grain weight

**Fig. 1.**
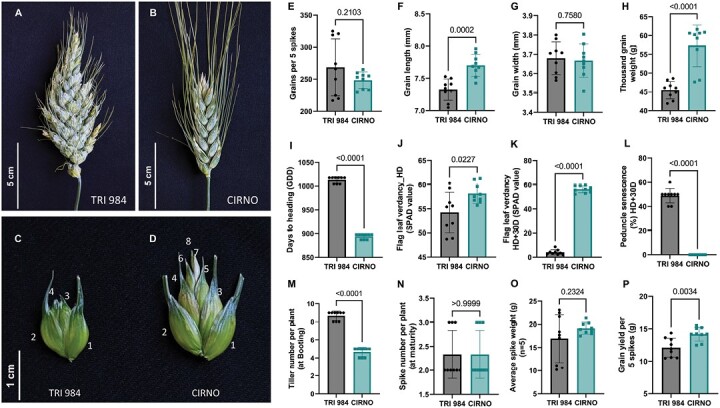
TRI 984 has a poor source–sink balance compared with CIRNO. (A, B) Spike-branching phenotype of TRI 984 (A) and standard spike of CIRNO (B). (C, D) Spikelet from the central part of the TRI 984 main rachis (C) shows reduced florets relative to a spikelet from a similar position in CIRNO (D). (E) Despite having more spikelets, there is no increase in grain number per five spikes in TRI 984. (F–H) The grains are smaller in TRI 984, leading to a reduction in thousand-grain weight. (I–L) TRI 984 exhibited delayed heading (I), lower flag leaf verdancy at heading (J), and accelerated flag leaf and peduncle senescence (K, L). (M–O) TRI 984 had more tillers at booting (M), but final spike number per plant remained unaltered (N) and there was no difference in spike weight (O). (P) Grain yield per five spikes was reduced in TRI 984 compared with CIRNO. An unpaired *t*-test was performed to determine significance in (E–P) and the resulting *P*-values (two-tailed analysis) are displayed. Data represented in (J, K, M, N) were obtained from greenhouse experiment, while the all remaining traits were phenotyped from field grown plants.

Consistent with previous findings using different germplasm (Wolde *et al.*, 2021), while the spike-branching landrace TRI 984 had more spikelets and florets per spike, the spikelets contained fewer florets and grains than the elite durum cultivar CIRNO (Fig. 1A–D; Supplementary Fig. S5A–C). However, we found no difference in grain number per five spikes, but a considerably reduced thousand-grain weight associated with shorter grains was observed in TRI 984 (Fig. 1E–H). While CIRNO flowered earlier (Fig. 1I), it had greener flag leaves both at heading (Fig. 1J) and after 30 d of heading (Fig. 1K) along with greener peduncles (Fig. 1L). Besides, CIRNO had longer but narrower flag leaves (Supplementary Fig. S5D, E), fewer tillers (Fig. 1M), yet spikes per plant remained unaltered (Fig. 1N), and it had shorter spikes (Supplementary Fig. S5F) and shorter plant stature (Supplementary Fig. S5G) compared with TRI 984. We found considerable differences in spike branching expressivity in TRI 984 (Supplementary Fig. S5H), which can explain the grain number, grain weight, and spike weight variations observed among various replications of TRI 984 (Fig. 1E–H, O, P). Such inconsistencies in spike branching have also been reported earlier in another Miracle-Wheat elite cultivar biparental population (Wolde *et al.*, 2021). Furthermore, there was no difference in the average spike weight (*n*=5) (Fig. 1O), but CIRNO had more grain yield per five spikes (Fig. 1P). These observations indicate a clear difference in terms of assimilate production and reallocation patterns between the two genotypes. Variations in tiller number (at booting) might indicate different resource partitioning strategies in TRI 984 and CIRNO during the pre-anthesis phase; however, there was no difference in the total number of spikes at maturity. Importantly, the spike-branching landrace TRI 984 exhibited a shorter grain filling period (quicker senescence), which implies reduced resource production and reallocation compared with CIRNO (delayed senescence) during the post-anthesis phase. Besides, the resources required to maintain the vegetative parts might be higher in the case of TRI 984 because of the taller plant architecture than CIRNO. Based on these observations, it is conceivable that genetic analysis of the corresponding landrace elite recombinants (TRI 984 × CIRNO) that vary in source–sink balance might provide mechanistic insights into the negative effect of spike branching on grain number per spikelet and grain weight, two major components of final grain yield.

### Grains per spikelet and thousand-grain weight are associated with senescence rate

**Fig. 2.**
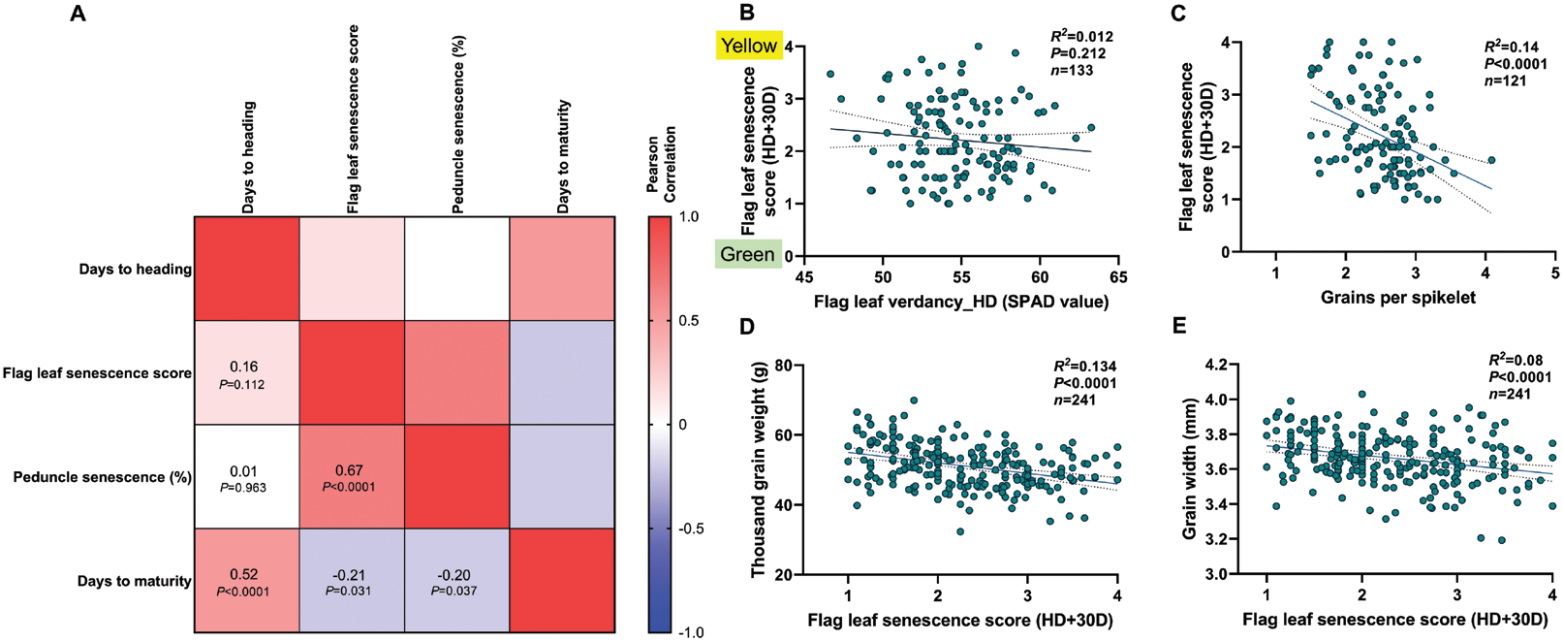
Functional ‘stay-green’ phenotype was observed in the landrace elite recombinants. (A) Flag leaf and peduncle senescence were positively related, independent of days to heading. (B) Flag leaf verdancy at the heading did not impact the progression of senescence. (C–E) Grains per spikelet was associated with flag leaf senescence rate (C) and the RILs with delayed senescence had higher thousand-grain weight (D), due to wider grains (E). (B–E) are linear regression plots with the explanatory variable on the *x*-axis, while the *y*-axis represents the response variable. *R*^2^ is the phenotypic variance explained, and the corresponding *P*-values of the regression analysis are displayed. The data presented in (A–E) were phenotyped from field-grown plants.

As expected, we witnessed a considerable diversity for all the plant and spike architectural traits (Supplementary Fig. S6). Importantly, flag leaf and peduncle senescence rates were independent of the heading date; this implies that there is a possibility for the lines that flowered late to senesce early and vice versa (Fig. 2A). The lines with delayed flag leaf senescence also had the tendency of retaining green/verdant peduncles for a longer duration (Fig. 2A). In addition, the intensity of flag leaf greening (SPAD meter value) at heading had no effect (*R*^2^=0.012, *P*=0.212) on the progress of senescence (scored at 30 d after heading), indicating that these traits are largely independent (Fig. 2B). Flag leaf length and delay in senescence were positively related (*R*^2^=0.045, *P*=0.0034), while flag leaf width did not influence these (Supplementary Fig. S7A, B). Moreover, we observed that the lines with more verdant/greener flag leaves at heading (higher SPAD value) also had a more significant number of florets per spikelet (*R*^2^=0.085, *P*=0.0014), in line with the expected consequence of source strength on sink organ establishment before anthesis (Supplementary Fig. S7C). Intriguingly, the number of florets and grains per spikelet, which was determined earlier, was somewhat associated with senescence rate, i.e. the lines with more florets and grains per spikelet tended to display delayed flag leaf senescence (*R*^2^=0.071, *P*=0.0007 and *R*^2^=0.14, *P*<0.0001) (Supplementary Fig. S7D; Fig. 2C). Likewise, it has been previously reported that higher grain number increased the post-anthesis radiation use efficiency in wheat (Reynolds *et al*., 2005). Here, we mapped a QTL on chromosome (Chr) 5A (*bh*^*t*^*-A3*) influencing grains per spikelet and flag leaf senescence rate (Supplementary Table S1). This possibly implies a gene/QTL-mediated pleiotropic association between sink number and flag leaf senescence rate, although these traits might not be physiologically dependent. Besides, the delayed senescence rate had a positive effect on thousand-grain weight (*R*^2^=0.13, *P*<0.0001) (Fig. 2D). We realized that the observed increase in thousand-grain weight is primarily due to the change in grain width (*R*^2^=0.08, *P*<0.0001) (Fig. 2E) and not grain length (Supplementary Fig. S7E), suggesting that grain width is more plastic, influenced by resource reallocation compared with grain length. Nevertheless, it is clear that the longer duration of green flag leaf and peduncle is not simply ‘cosmetic’—it influences grain yield determinants. This vital evidence supports our hypothesis that dissecting the source–sink relationship might have relevance in balancing the trade-offs that negatively regulate the final grain yield in ‘Miracle-Wheat’-like genotypes.

### The bh^t^-A1 locus underlies sink and source capacity

**Fig. 3.**
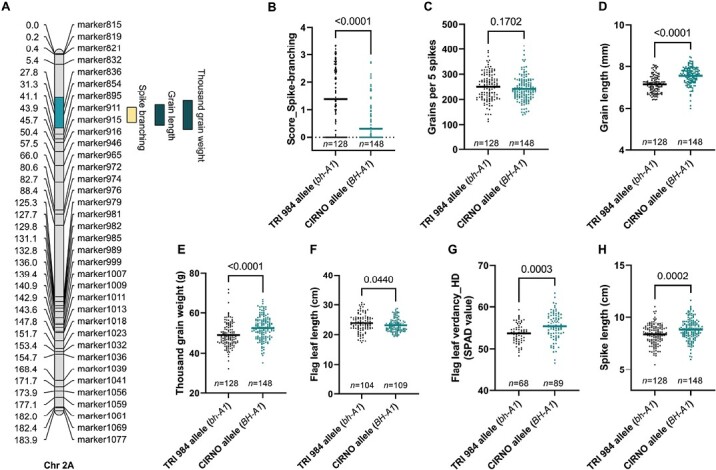
*bh* ^*t*^ *-A1* induces spike branching but with a grain weight trade-off. (A) A major effect QTL hotspot for spike branching, grain length, and weight was mapped on the short arm of Chr 2A. (B–H) RILs with the TRI 984 allele showed spike branching (B), no difference in grains per five spikes (C), but a reduction in grain length (D), thousand-grain weight (E), flag leaf length (F), flag leaf verdancy at heading (G), and spike length (H). In (B–H), *n* represents the number of RILs that were compared for each allele class; TRI 984 allele data points are in black and CIRNO allele data points are in cyan. An unpaired *t*-test was used to determine the statistical significance, and the resulting *P*-values (two-tailed analysis) are displayed for all the comparisons. Data obtained from field grown plants were used for the analysis in (A–H).

Using a scoring method based on the phenotype of the spike (Supplementary Fig. S3), we mapped a major effect QTL for spike branching on Chr 2A (Fig. 3A, Supplementary Table S1) associated with the previously known locus *bh*^*t*^*-A1* (Poursarebani *et al*., 2015). Regardless of the increase in spikelet number per spike owing to the lateral branching (Fig. 3B), there was no difference in the total grains per spike (Fig. 3C) but the *bh*^*t*^*-A1* locus was associated with a reduction in grain length (Fig. 3D) and thousand-grain weight (Fig. 3E). While the *bh-A1* lines had slightly longer flag leaf blades (Fig. 3F), the flag leaf verdancy at heading (Fig. 3G) and spike length (Fig. 3H) were negatively affected. Thus, the TRI 984 allele induced spike branching and in addition might also affect the source capacity. Eventually, there was no advantage in the final grain yield because of spike branching.

### bh^t^-A3, a novel spike-branching locus on chromosome 5A, reshapes source–sink dynamics

**Fig. 4.**
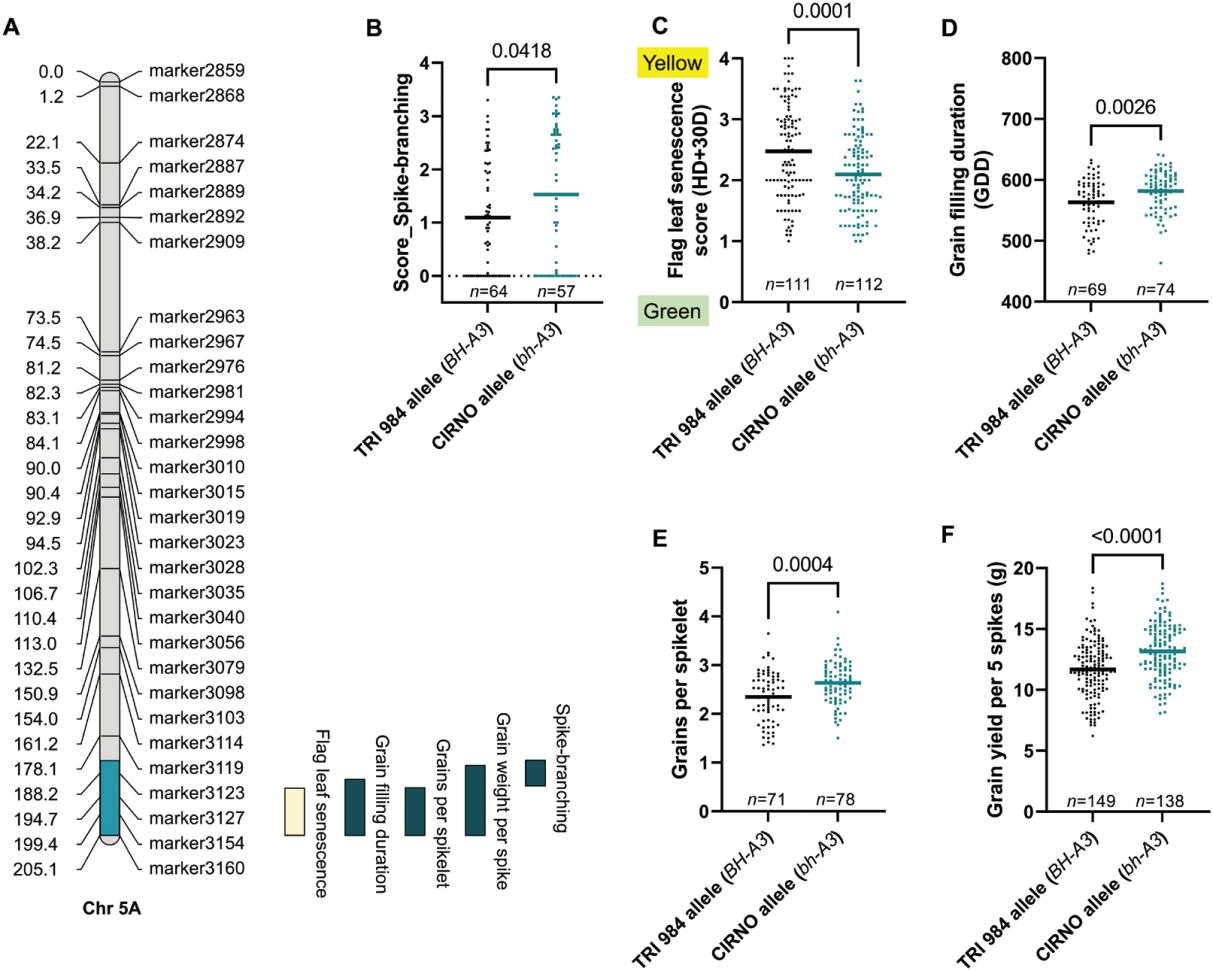
*bh* ^*t*^ *-A3*, a new modifier locus for spike branching. (A) *bh*^*t*^*-A3* mediates spike branching, flag leaf senescence rate, grain filling duration, grains per spikelet, and grain yield per spike. (B–F) The CIRNO allele increases the expressivity of spike branching (when *bh*^*t*^*-A1* is present) (B), delays flag leaf senescence rate (C), increases grain filling duration (D), grains per spikelet (E), and grain yield per five spikes (F). In (B–F), *n* represents the number of RILs that were compared for each allele class; TRI 984 allele data points are in black and CIRNO allele data points are in cyan. An unpaired *t*-test was used to determine the statistical significance, and the resulting *P*-values (two-tailed analysis) are displayed for all the comparisons. Data obtained from field grown plants were used for the analysis in (A–F).

Following similar phenotyping (Supplementary Fig. S3), we mapped a QTL for spike branching on the long arm of Chr 5A; here the CIRNO allele contributed to the spike-branching phenotype (Fig. 4A; Supplementary Table S1). We named the newly identified spike-branching modifier locus as ‘*bh*^*t*^*-A3*’ following the previously known *bh*^*t*^*-A1* (Poursarebani *et al.*, 2015) and *bh*^*t*^*-A2* (Wolde *et al.*, 2021) loci. Interestingly, the spike-branching effect of the *bh*^*t*^*-A3* locus manifests only in the presence of the mutated *bh*^*t*^*-A1* allele (Fig. 4A; Supplementary Fig. S8A–D; Supplementary Table S1). We divided the RILs into two sub-groups for QTL mapping, viz. by fixing (i) *bh*^*t*^*-A1* and (ii) *BH*^*t*^*-A1*, and the outcome confirmed the epistasis of the *bh*^*t*^*-A3* to *bh*^*t*^*-A1* locus (Supplementary Fig. S8A). Possibly, this indicates that the plasticity for spike branching is introduced by *bh*^*t*^*-A1*, i.e. it might be first essential to have *bh*^*t*^*-A1* to disrupt the spikelet meristem identity and only then might the *bh*^*t*^*-A3* locus modify the branching intensity in the spikes. Besides, the grain number increase was only observed in the spike-branching RILs—when *bh*^*t*^*-A1* is present (Supplementary Fig. S8C). Moreover, in this region, we found co-localized QTLs for an array of traits influencing source–sink dynamics. The CIRNO allele was associated with spike branching (Fig. 4B), delayed flag leaf senescence (Fig. 4C), more extended grain filling period (Fig. 4D), increased grains per spikelet (Fig. 4E), and grain yield per five spikes (Fig. 4F). Besides, we also found a subtle, yet positive, effect on grain width (Supplementary Fig. S9A), florets per spikelet (Supplementary Fig. S9B), straw biomass (Supplementary Fig. S9C), and harvest index (Supplementary Fig. S9D); but days to heading was not affected (Supplementary Fig. S9E). Interestingly, we found that the observed variations in flag leaf senescence, grain filling duration, and grain width were not dependent on the presence of *bh*^*t*^*-A1* (Supplementary Fig. S8E–G). This pattern implies that the phenotypic variation explained by the 5A QTL hotspot for spike branching expressivity and senescence rate might be the outcome of at least two linked genes. Taken together, this trend suggests that the favourable CIRNO allele (*bh*^*t*^*-A3*) mediates enhanced assimilate production and reallocation of the resources to sink organs, including the lateral branches/supernumerary spikelets because of longer grain filling duration.

### GPC-B1 is the major determinant of senescence rate and thousand-grain weight

**Fig. 5.**
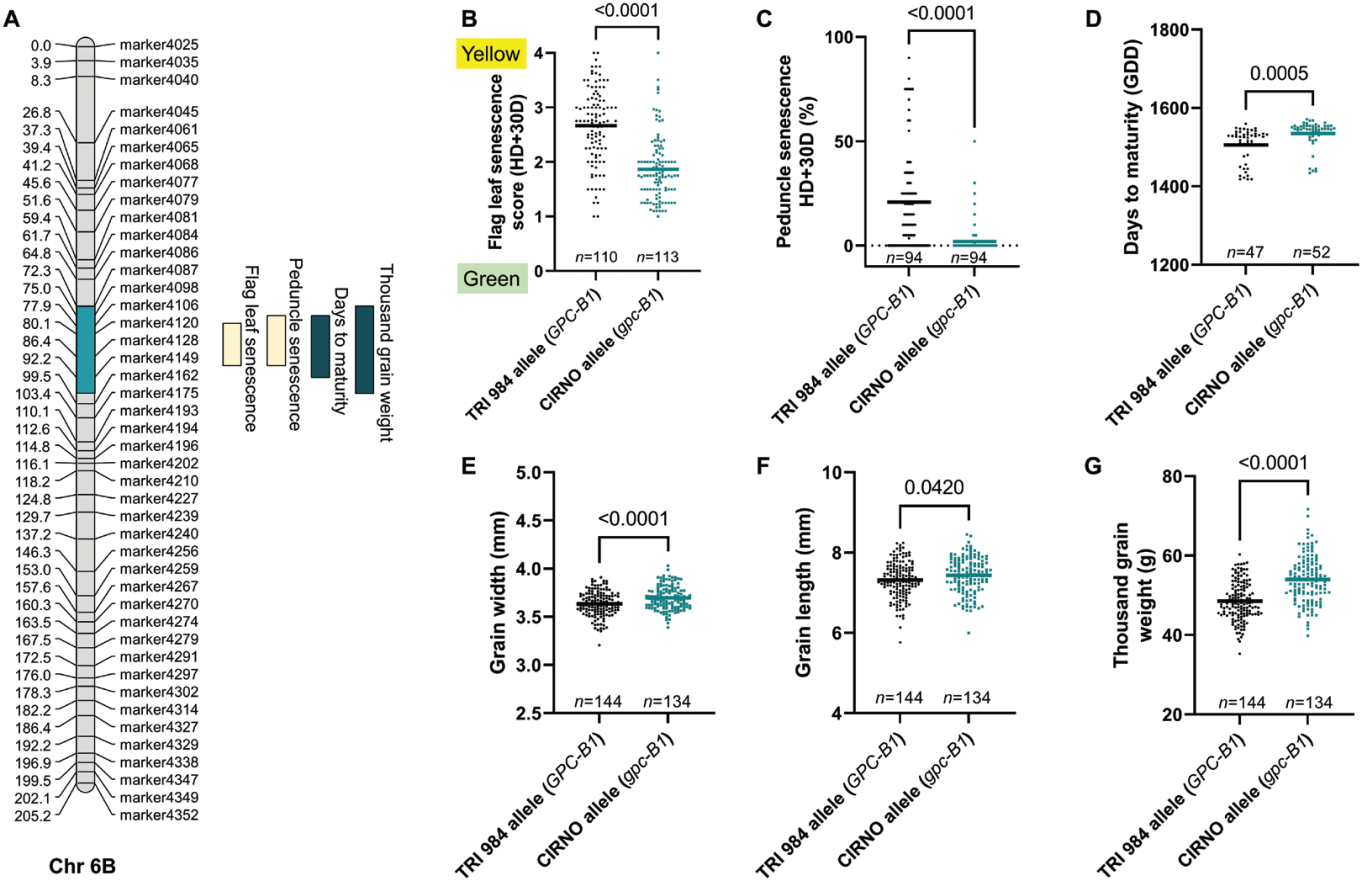
*gpc-B1* regulates senescence rate and grain weight. (A) QTLs for overall senescence rate and thousand-grain weight co-localized on Chr 6B. (B–D) The modern (CIRNO) allele mediated delay in flag leaf senescence (B), peduncle senescence (C), and days to maturity (spike senescence) (D). (E–G) The resulting increase in the post-anthesis phase is translated into an increase in grain width (E), grain length (F), and eventually thousand-grain weight (G). In (B–G), *n* represents the number of RILs that were compared for each allele class; TRI 984 allele data points are in black and CIRNO allele data points are in cyan. An unpaired *t*-test was used to determine the statistical significance, and the resulting *P*-values (two-tailed analysis) are displayed for all the comparisons. Data obtained from field grown plants were used for the analysis in (A–G).

A QTL on Chr 6B, which most likely is associated with *GPC-B1* (Uauy *et al.*, 2006), explained most of the observed phenotypic variance for the overall plant senescence rate (Fig. 5A; Supplementary Table S1). Likewise, it was found that mutations in the NAC domain of *NAM-A1* (*GPC-A1*) delayed peduncle and flag leaf senescence (Harrington *et al.*, 2019). In the current study, the CIRNO allele ensured delay in the flag leaf (Fig. 5B), peduncle (Fig. 5C), and spike senescence (days to maturity) (Fig. 5D). Therefore, there might be a possibility of more reallocation into the sink organs, leading to an increase in grain width (Fig. 5E) and grain length (Fig. 5F). Accordingly, we observed a considerably higher thousand-grain weight in the RILs that senesce late (Fig. 5G). But, there was no meaningful difference in grain number per five spikes (Supplementary Fig. S10A), straw biomass (Supplementary Fig. S10B), and harvest index (Supplementary Fig. S10C). Although flag leaf length was not directly affected by this QTL (Supplementary Fig. S10D), longer flag leaves, in general, had a positive effect on thousand-grain weight in both allele groups (Supplementary Fig. S11A, B), viz. *GPC-B1* (*R*^2^=0.11, *P*=0.0003) and *gpc-B1* (*R*^2^=0.043, *P*=0.041). Notably, the relationship between flag leaf length and grain weight was relatively stronger in the early senescing genotypes. In fact, the contribution to thousand-grain weight per unit length of flag leaf was higher in the case of the *gpc-B1* allele that is associated with delayed senescence (Supplementary Fig. S11C). Furthermore, we also included the effect of the allelic status at *bh*^*t*^*-A1*, another major QTL for grain weight, in addition to *gpc-B1*. Here, the RILs with various allele combinations revealed a similar positive relationship between flag leaf length and grain weight (Supplementary Fig. S11D–G), viz. *BH*^*t*^*-A1*+*gpc-B1* (*R*^2^=0.113, *P*=0.0118), *BH*^*t*^*-A1*+*GPC-B1* (*R*^2^=0.164, *P*=0.0032), *bh*^*t*^*-A1*+*gpc-B1* (*R*^2^=0.12, *P*=0.0586), and *bh*^*t*^*-A1*+*GPC-B1* (*R*^2^=0.136, *P*=0.0048). Overall, these observations support the idea of the importance of source strength, i.e. possibly more resource production and reallocation (delayed senescence) enhanced thousand-grain weight in both spike-branching and standard non spike-branching genotypes evaluated in the current population.

### Specific additive and epistatic interactions may increase yield potential in spike-branching genotypes

**Fig. 6.**
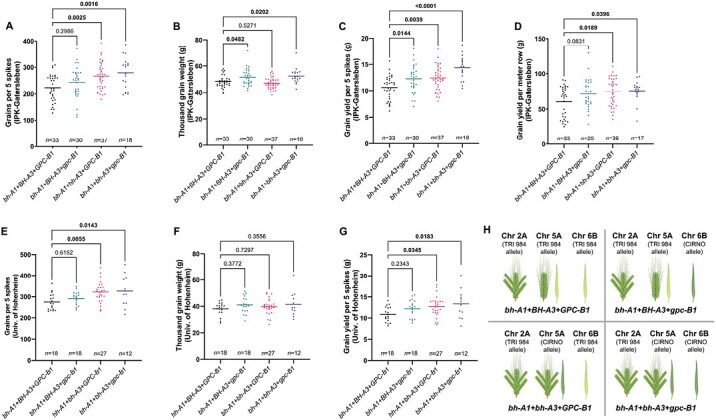
*bh* ^*t*^ *-A1*, *bh*^*t*^*-A3*, and *gpc-B1* balance the grain yield trade-offs in spike-branching recombinants. The RILs with various combinations of alleles were phenotyped at IPK-Gatersleben (2021 and 2022) and the University of Hohenheim (2022). (A–D) At IPK, the spike-branching RILs that senesce late (*bh*^*t*^*-A1+bh*^*t*^*-A3+gpc-B1*) had higher grain number per five spikes (A), increased thousand-grain weight (B), and more grain yield per five spikes (C), and finally grain yield per metre row was also higher in the stay-green spike-branching RILs (calculated only at IPK) (D). (E–G) Likewise, at Hohenheim, we observed more grains per five spikes (E), but no change in thousand-grain weight (F), and eventually there was an increase in grain yield per spike (G). (H) Pictorial depiction of the various allelic combinations that are analysed in (A–G). In (A–G), one-way ANOVA followed by Dunnett’s test was used to determine the statistical significance. All the comparisons are made with respect to the *bh*^*t*^*-A1+BH*^*t*^*-A3+GPC-B1* allelic combination, and the corresponding *P*-values are displayed in all the graphs (significant ones are in bold). Data obtained from field grown plants were used for the analysis in (A–G). The image in (H) was partly created using BioRender.com. *bh*^*t*^*-A1+BH*^*t*^*-A3+GPC-B1*: recombinants with one locus for spike branching and two loci for accelerated senescence; *bh*^*t*^*-A1+BH*^*t*^*-A3+gpc-B1*: recombinants with one locus each for spike branching and delayed senescence; *bh*^*t*^*-A1+bh*^*t*^*-A3+GPC-B1*: recombinants with two loci for spike branching, while carrying one locus for delayed senescence; *bh*^*t*^*-A1+bh*^*t*^*-A3+gpc-B1*: recombinants with two loci each for spike branching and delayed senescence.

**Fig. 7.**
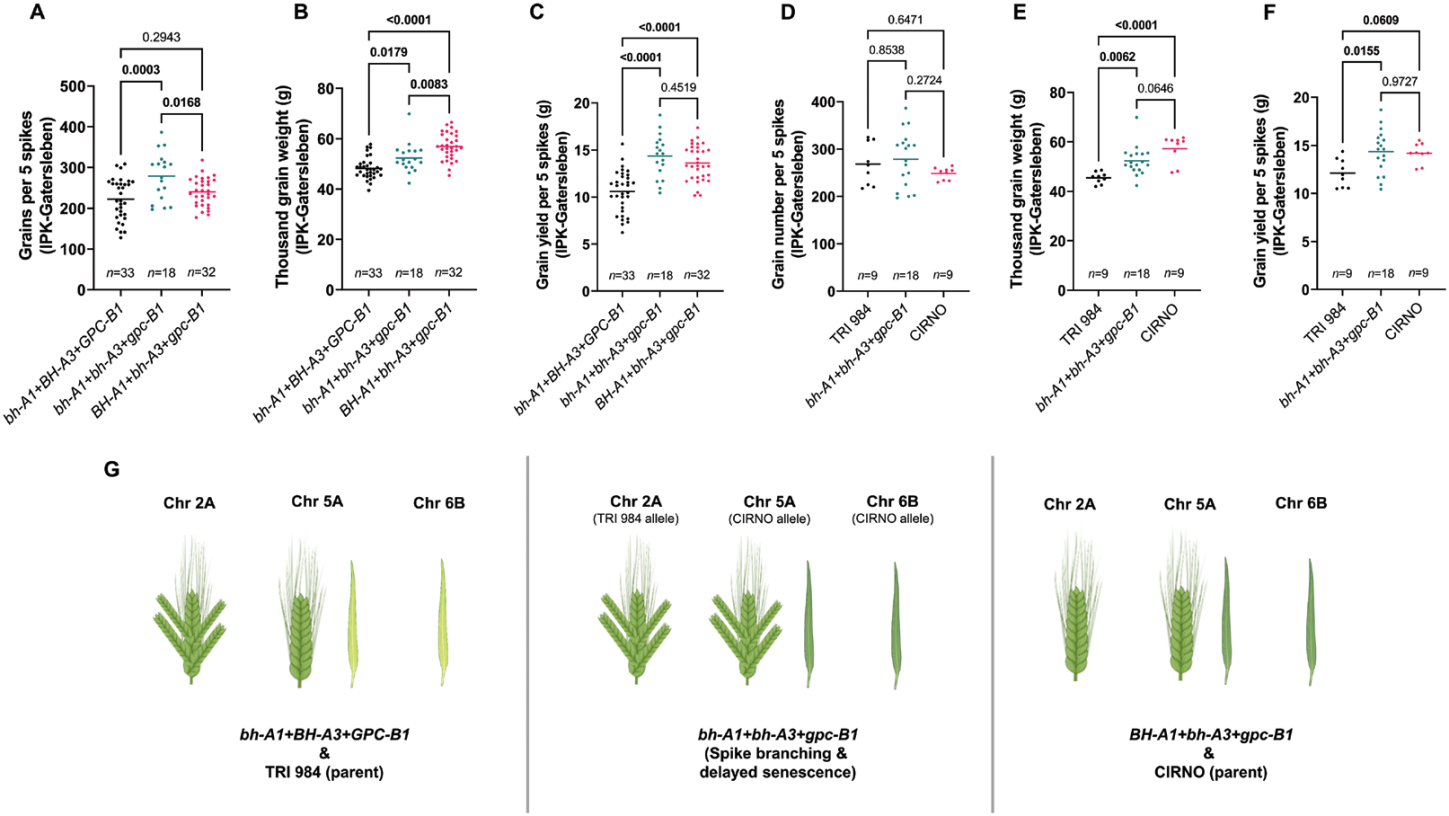
The favourable spike-branching RILs (*bh*^*t*^*-A1+bh*^*t*^*-A3+gpc-B1*) performed better than TRI 984, but similar to CIRNO. (A, B) The spike-branching RILs with delayed senescence produced more grains per spike (A), but with a reduction in thousand-grain weight than the corresponding genotypes with standard spike (B), i.e. no spike branching (*BH*^*t*^*-A1+bh*^*t*^*-A3+gpc-B1*). (C) Further, the spike grain yield was similar among the genotypes that senesce late, irrespective of the presence or absence of spike branching. (D) However, there was no difference in grain number compared with TRI 984 and CIRNO. (E) The favourable spike-branching genotypes had higher thousand-grain weight than TRI 984, whereas there was a reduction compared with CIRNO. (F) Finally, the spike grain yield was higher than TRI 984, but similar to CIRNO. (G) pictorial depiction of the various allelic combinations that are analysed in (A–F). In (A–F), one-way ANOVA followed by Dunnett’s test was used to determine the statistical significance. Multiple range comparison was performed, and the corresponding *P*-values are displayed in all the graphs (significant ones are in bold). Data obtained from field grown plants were used for the analysis in (A–F). The image in (G) was partly created using BioRender.com. *bh*^*t*^*-A1+BH*^*t*^*-A3+GPC-B1*: recombinants with one locus for spike branching and two loci for accelerated senescence; *bh*^*t*^*-A1+bh*^*t*^*-A3+gpc-B1*: recombinants with two loci each for spike branching and delayed senescence; *Bh*^*t*^*-A1+bh*^*t*^*-A3+gpc-B1*: recombinants without any spike-branching alleles, but containing two loci for delayed senescence.

**Fig. 8.**
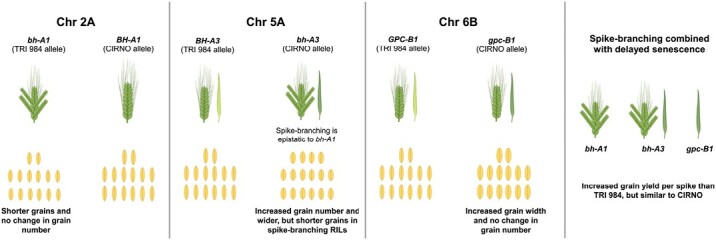
Summary of the physiological and genetic basis of interaction among *bh*^*t*^*-A1*, *bh*^*t*^*-A3*, and *gpc-B1* in the current landrace-elite recombinant population. The image was partly created using BioRender.com.

As the QTLs on Chr 2A, 5A, and 6B explain variations in key source–sink attributes, we analysed their various allelic combinations to better understand the grain yield trade-offs in spike-branching genotypes (Figs 6A–H, 7A–G, 8). Interestingly, the spike-branching lines carrying *bh*^*t*^*-A1* and *bh*^*t*^*-A3* loci along with *gpc-B1* had higher grain number per five spikes (Fig. 6A, E) and were associated with a delay in post-anthesis flag leaf senescence. However, the difference in thousand-grain weight was observed only at IPK (Fig. 6B; Supplementary Fig. S12A, B), while this effect was absent in Hohenheim (Fig. 6F; Supplementary Fig. S12C, D). Nevertheless, they had higher grain yield per five spikes (Fig. 6C, G) across all the three environments, viz. IPK-2021, IPK-2022, and University of Hohenheim-2022, compared with the early senescing branched spike RILs (*bh*^*t*^*-A1+BH*^*t*^*-A3+GPC-B1*). Moreover, grain yield (per metre row) was also higher in the stay-green spike-branching RILs than the ones that senesced early (Fig. 6D).

Next, we compared the impact of delayed senescence between the spike-branching RILs and those with standard spikes (no spike branching). Although, the spike-branching RILs carrying the favourable alleles (*bh*^*t*^*-A1*+*BH*^*t*^*-A3*+*GPC-B1*) had higher grain number (Fig. 7A), they had reduced thousand-grain weight (Fig. 7B) than the lines with standard spikes having similar senescence rate, i.e. *BH*^*t*^*-A1*+*bh*^*t*^*-A3*+*gpc-B1*; nevertheless, the final spike grain yield (Fig. 7C) was similar in both these cases. Finally, we compared the performance of these spike-branching RILs with both the parental lines. Here, we observed a non-significant increase in grain number, but a slightly reduced thousand-grain weight than CIRNO (Fig. 7D, E). However, there was an increase in thousand-grain weight and no change in grain number when compared with TRI 984 (Fig. 7D, E). Eventually, the grain yield per spike of the favourable spike-branching RILs was higher than TRI 984 and similar to CIRNO (Fig. 7F).

## Discussion

Over the course of domestication and breeding, grain yield determinants such as grain number and grain weight, but also grain quality traits under both favourable and stressful conditions, were the primary selection targets in all major cereal crops, including wheat (Voss-Fels *et al.*, 2019; McSteen and Kellogg, 2022). For instance, the selection of the semi-dwarf *Rht-1* allele was a vital driver of the ‘green revolution’ in wheat (Peng *et al.*, 1999); likewise, the prevalence of the less functional *GNI-A1* allele enabled higher floret fertility in the modern wheat cultivars (Golan *et al.*, 2019; Sakuma *et al.*, 2019). However, substantial genetic yield gaps (the difference between the genetic yield potential of a crop in a particular environment and the potential yield of the current local cultivar) suggest the presence of untapped genetic diversity for enhancing wheat grain yield (Senapati *et al.*, 2022). Grain yield can be optimized by fine-tuning various developmental processes (Mathan *et al.*, 2016) and introducing ‘drastic variations’ in crop breeding (Abbai *et al.*, 2020). The genetic pathways that coordinate inflorescence architecture are dissected in staple grasses (Kellogg, 2022; Koppolu *et al.*, 2022), which might have relevance for minimizing the genetic yield gap. Here, we considered the case of spike-branching Miracle-Wheat as a potential option for increasing sink strength (more spikelets and grains per spike). However, the genetic analysis of the TRI 984 × CIRNO recombinants revealed a couple of significant limitations.

First, we recorded inconsistencies in the expressivity (degree) of spike branching (in the RILs that carried similar QTLs/alleles; Figs 3B, 4B; Supplementary Fig. S8B). Likewise, large variations in spike-branching intensity (Supplementary Fig. S5H) and eventually grain number per spike (Fig. 1E) were also observed in TRI 984. Wolde *et al.* (2021) reported that the expressivity of spike branching in a particular genotype was higher in the outer rows compared with the inner rows of the plot. However, no new QTLs were mapped that specifically explained such differences. One explanation might be that field-grown plants experience competition for various resources, including light (Huber *et al.*, 2021; Postma *et al.*, 2021), especially in the inner rows (Rebetzke *et al.*, 2014). Furthermore, we had two genotypes (three rows each) in 1.5 m^2^ plots in the current study and the neighbouring genotype was not the same in all the three replications, which might have affected spike branching differently due to asymmetric plant–plant competition. In this regard, future studies investigating the response of various source and sink component traits in high-density monoculture plots or simulated canopy shade (Golan *et al.*, 2022) are required to uncover the genetic framework of plant–plant competition and its effect on spike branching expressivity.

Next, the spike-branching induced trade-off on grain weight was another major concern. Previously, Poursarebani *et al*. (2015) reported that the *bh*^*t*^*-A1* locus increases grain number, but with a grain weight trade-off. Likewise, we also observed considerably smaller grains in the spike-branching genotypes (Figs 1F, 3D). However, in the current study, the spike-branching phenotype induced by *bh*^*t*^*-A1* had no effect on the final grain number (Figs 1E, 3C). Interestingly, there was no thousand-grain weight trade-off in the spike-branching Bellaroi × TRI 19165 semi-dwarf RILs (Wolde *et al.*, 2021) and also in the Floradur NILs with supernumerary spikelets (Wolde *et al.*, 2019), thus warranting the analysis of source–sink dynamics in the non-canonical spike forms. Here, it is vital to emphasize the relevance of the post-anthesis (yield realization) events, chiefly related to the transfer of assimilates to the previously established sink organs during grain filling (Murchie *et al.*, 2023; Slafer *et al.*, 2023). In this context, the senescence rate might have an impact on grain filling duration (Kichey *et al.*, 2007; Christopher *et al.*, 2016; Chapman *et al.*, 2021b; Hassan *et al.*, 2021; Li *et al.*, 2022), i.e. extended photosynthesis leading to more assimilate production and allocation to the developing grains. However, final grain weight was not strongly related with starch/sugar levels or the corresponding enzymatic capacity in 54 diverse wheat genotypes, but it might be a function of early developmental events (Fahy *et al.*, 2018). In the current study, we found that higher grain number per spikelet (Fig. 2C) and grain weight (Fig. 2D) is associated with delayed flag leaf, peduncle, and spike senescence. As expected, the observed effect of senescence rate might be because of the differences in various sink strength-related traits such as spikelet number per spike (spike branching) (Fig. 3B), and floret number per spikelet (Supplementary Fig. S9B) in our RIL population. As the sink strength increased, perhaps the extended photosynthetic period was meaningful for influencing the final grain yield. This trend further establishes the rationale for understanding the genetic and molecular framework of source and sink-related component traits to enable grain yield gains (Brinton and Uauy, 2019; Reynolds *et al.*, 2022). With this, the favourable alleles explaining the source–sink dynamics might assist in improving grain number and grain weight in the spike-branching genotypes. Here, we analysed the interactions among *bh*^*t*^*-A1*, *bh*^*t*^*-A3*, and *gpc-B1*; the *bh*^*t*^*-A1* and *bh*^*t*^*-A3* loci regulated spike branching, but also source strength, while *gpc-B1* delayed senescence rate and increased thousand-grain weight (Figs 6, 7). Transcriptional analysis of WT and *NAM* (*GPC*) RNAi lines revealed differential regulation of genes related to various processes, including photosynthesis and nitrogen metabolism, during flag leaf senescence (Andleeb *et al.*, 2022). Our preliminary genetic evidence indicates that *gpc-B1* might function independently of the spike-branching-associated loci (Supplementary Fig. S13A, B). In any case, as speculated, the spike-branching RILs with an extended photosynthetic period (delayed senescence) had considerably higher grain yield (per metre row) compared with branched spike genotypes that senesced early (Fig. 6D). In this case, the stay-green spike-branching RILs had about 11 additional grains per spike (SEM: ±3.17) (Fig. 6A). However, we believe that the grain number difference might be due to the interaction between floret number and flag leaf senescence, which is mediated by the *bh*^*t*^*-A3* locus; the CIRNO allele increased florets per spikelet (Supplementary Fig. S9B) and delayed flag leaf senescence (Fig. 4C). The pre-anthesis floret degeneration and post-anthesis flag leaf senescence might share a common genetic basis thereby primarily affecting the tip of the respective organs, i.e. spikelet meristem/rachilla and flag leaf, respectively. Therefore, it is conceivable that the underlying gene might have a pleiotropic effect on floret degeneration and flag leaf senescence, thus explaining the grain number difference. Recently, enrichment of senescence-related transcripts has been reported during pre-anthesis tip degeneration in barley spikes (Shanmugaraj *et al*., 2023), which further supports our hypothesis. Then in our field experiments conducted at IPK-Gatersleben (2021 and 2022), we found an 8.5% (SEM: ±3.11%) increase in average grain weight (Fig. 6B) in the spike-branching genotypes that senesce late. The 2.53% (SEM: ±1.01%) rise in grain width (Supplementary Fig. S12A) majorly contributed to the grain weight difference, as the grain length remained unaffected (Supplementary Fig. S12B). Incidentally, it was found that grain width increased during wheat evolution under domestication (Gegas *et al.*, 2010). Besides, it might be interesting to evaluate the effect of expansin genes in the spike-branching lines as the ectopic expression of *TaExpA6* increased grain length (Calderini *et al.*, 2021).

Further, we would like to emphasize certain limitations in our experimental set-up: we used relatively small plots (only 1.5 m^2^) with two genotypes in one plot; therefore, the influence of the border effect (Rebetzke *et al.*, 2014) cannot be excluded in grain yield per row calculations and besides, the evaluated population are landrace elite recombinants, which might create another bias in the observed yield increase. Although there is a significant increase in grain number per five spikes and grain weight in the stay-green spike-branching recombinants, the actual yield advantage might be better understood by evaluating the effect in isogenic backgrounds (NILs) and larger plots in multiple environments. In this context, we are developing spike-branching CIRNO NILs for these follow-up experiments. Another trade-off associated with extending the grain filling duration that is not addressed here is its likely impact on grain nutrition profile; the functional *NAM-B1* allele improves grain protein, iron, and zinc content by accelerating the senescence process (Uauy *et al.*, 2006). Then, the status of the stay-green spike-branching RILs under unfavourable conditions is also beyond the scope of the current study; however, previous reports indicate a positive effect of stay-green phenotypes on wheat grain yield under drought and heat (Lopes and Reynolds, 2012). Similarly, delay in senescence led to higher grain number and tiller number but lower thousand-grain weight under nitrogen-limiting conditions (Derkx *et al.*, 2012). In addition, a recent simulation study indicates the advantage of cultivating late-maturing wheat varieties in future climate scenarios (Minoli *et al.*, 2022), suggesting that a delay in senescence rate might eventually be beneficial.

### Conclusion

The physiological and genetic analysis of TRI 984 × CIRNO recombinants revealed that (i) extended verdant flag leaf, peduncle, and spike led to higher grain yield per spike as the traits influencing sink strength segregated, including spike branching; (ii) three QTL regions—on Chr 2A (*bh*^*t*^*-A1*), Chr 5A (*bh*^*t*^*-A3*), and Chr 6B (*gpc-B1*)—were associated with source–sink strength in the current biparental population; and (iii) an increase in grain number and grain weight is predominantly possible among the stay-green, spike-branching genotypes. Finally as wheat grain yield is also sink-limited, we propose that introducing spike branching as a breeding target might enable advancing genetic gains while minimizing the gap between genetic yield potential and the actual realized yield. Although we provide insights into the genetic basis of grain yield determination in ‘Miracle-Wheat’, it is still necessary to understand the basis of inconsistencies in the degree of spike branching within the same genotype but also in diverse genetic backgrounds. To achieve this, tracking the source-strength dynamics during the early developmental stages might be necessary.

## Supplementary data

The following supplementary data are available at *JXB* online.

Fig. S1. The RIL population was genotyped using the 25K array.

Fig. S2. Tracking the progression of senescence rate 30 d after heading.

Fig. S3. Dosage-based scoring method (0–4 point scale) for spikes with supernumerary spikelets and spike-branching phenotypes.

Fig. S4. Validation of Marvin results.

Fig. S5. TRI 984 vs CIRNO.

Fig. S6. Phenotypic distribution of various plant and spike architectural traits across the RIL population.

Fig. S7. Relationship between various traits across the population.

Fig. S8. Spike-branching phenotype explained by *bh-A3* being epistatic with *bh-A1*.

Fig. S9. *bh-A3* influences grain width, florets per spikelet, straw biomass, and harvest index, and has no effect on days to heading.

Fig. S10. *gpc-B1* does not influence grains per five spikes, straw biomass, harvest index, and flag leaf length.

Fig. S11. Effect of flag leaf length and thousand-grain weight in RILs.

Fig. S12. Genetic interaction among *bh-A1*, *bh-A3*, and *gpc-B1*.

Fig. S13. The flag leaf senescence rate of various allele combinations revealed that *gpc-B1* is independent of *bh-A1* and *bh-A3* and *gpc-B1* exhibit additive effect.

Table S1. Summary of the mapped QTLs and their attributes.

Dataset S1. Source data for main Figs 1–7.

Dataset S2. Source data for Supplementary Figs S4, S5, and Supplementary Figs S7–S13.

erad373_suppl_Supplementary_Dataset_S1

erad373_suppl_Supplementary_Dataset_S2

erad373_suppl_Supplementary_Figures_S1-S13

erad373_suppl_Supplementary_Tables_S1

## Data Availability

Supplementary Dataset S1 (48 excel sheets arranged figure-wise) and Supplementary Dataset S2 (43 excel sheets arranged figure-wise) contain the data used for testing statistical significance for all the corresponding main and supplementary figures respectively. The phenotypic trait means (field experiments at IPK-Gatersleben) and genotypic data of the mapping population are deposited in e!DAL (Arend *et al.*, 2014) and can be accessed at: http://dx.doi.org/10.5447/ipk/2023/17.

## Abbreviations

Chr: chromosome
RIL: recombinant inbred line
QTL: quantitative trait locus
